# The current status and future of targeted-immune combination for hepatocellular carcinoma

**DOI:** 10.3389/fimmu.2024.1418965

**Published:** 2024-08-05

**Authors:** Liyuan Hao, Shenghao Li, Fanghang Ye, Hengyi Wang, Yuxin Zhong, Xiaoyi Zhang, Xiaoyu Hu, Xiaopeng Huang

**Affiliations:** ^1^ Clinical Medical College, Chengdu University of Traditional Chinese Medicine, Chengdu, Sichuan, China; ^2^ Department of Infectious Diseases, Hospital of Chengdu University of Traditional Chinese Medicine, Chengdu, Sichuan, China; ^3^ Department of Urology/Andrology, Hospital of Chengdu University of Traditional Chinese Medicine, Chengdu, Sichuan, China

**Keywords:** HCC, ICIS, targeted-immune combination, anti-PD-1, anti-PD-L1, CTLA-4

## Abstract

Hepatocellular carcinoma (HCC) is one of the most common cancers and the third leading cause of death worldwide. surgery, transarterial chemoembolization (TACE), systemic therapy, local ablation therapy, radiotherapy, and targeted drug therapy with agents such as sorafenib. However, the tumor microenvironment of liver cancer has a strong immunosuppressive effect. Therefore, new treatments for liver cancer are still necessary. Immune checkpoint molecules, such as programmed death-1 (PD-1), programmed death-ligand 1 (PD-L1), and cytotoxic T lymphocyte antigen-4 (CTLA-4), along with high levels of immunosuppressive cytokines, induce T cell inhibition and are key mechanisms of immune escape in HCC. Recently, immunotherapy based on immune checkpoint inhibitors (ICIs) as monotherapy or in combination with tyrosine kinase inhibitors, anti-angiogenesis drugs, chemotherapy agents, and topical therapies has offered great promise in the treatment of liver cancer. In this review, we discuss the latest advances in ICIs combined with targeted drugs (targeted-immune combination) and other targeted-immune combination regimens for the treatment of patients with advanced HCC (aHCC) or unresectable HCC (uHCC), and provide an outlook on future prospects. The literature reviewed spans the last five years and includes studies identified using keywords such as “hepatocellular carcinoma,” “immune checkpoint inhibitors,” “targeted therapy,” “combination therapy,” and “immunotherapy”.

## 1 Introduction

**Figure 1:**
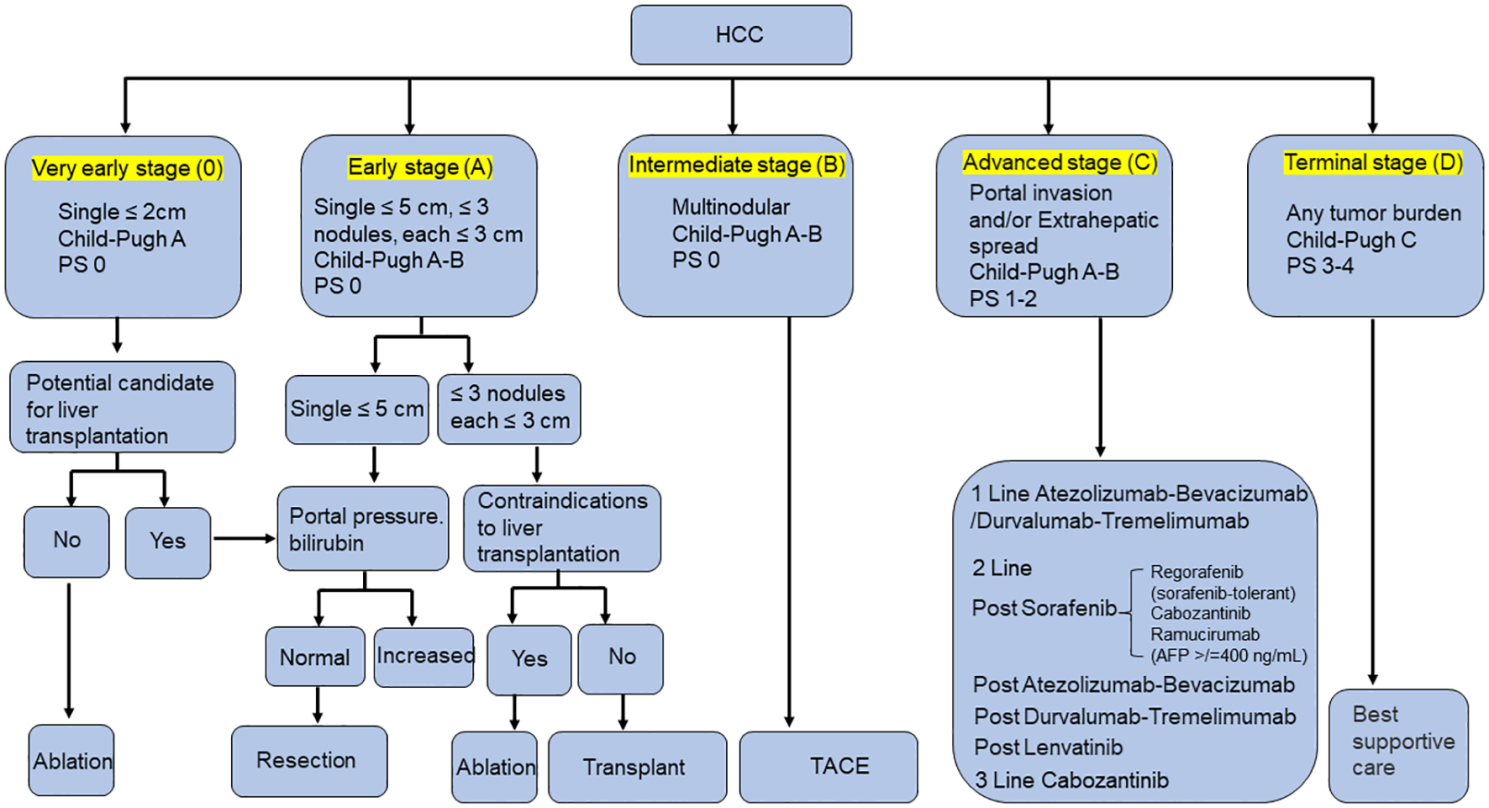
Overview of BCLC staging and treatment strategy in HCC.

Liver cancer is the sixth most common malignancy and the third leading cause of cancer-related death worldwide (1). Currently, hepatocellular carcinoma (HCC) accounts for about 75-85% of primary liver cancer, and is among the most common malignant tumors, posing a serious threat to public health (2). The Barcelona Clinic Liver Cancer (BCLC) system is the most commonly recommended staging system for HCC. Based on the underlying liver function, as assessed by the Child–Pugh score, and the performance status, HCC patients can be classified into BCLC stage 0, A, B, C and D (3). Most clinical practice guidelines recommend excision, ablation, and transplantation for patients with early HCC (BCLC 0, A). For patients with intermediate (BCLC B) and advanced (BCLC C) HCC, preferred treatments include transcatheter arterial chemoembolization (TACE), systemic therapy, local ablation therapy, radiotherapy and targeted drug therapy with sorafenib (**Figure 1**) (4–8). However, the treatment of advanced HCC (aHCC) remains inadequate. The tumor’s propensity for invasion, metastasis and recurrence results in low overall survival (OS), high mortality and poor prognosis.

HCC is a chronic inflammatory cancer that expresses multiple antigens and mediates immune responses. In recent years, immunotherapy has shown beneficial results in HCC (9). Immune checkpoint inhibitors (ICIs) therapy, especially antibodies targeting the programmed death-1 (PD-1)/programmed death-ligand 1 (PD-L1) pathway, represents a major breakthrough in oncology drug development over the past decade. ICIs exerts anti-tumor effects by blocking the interaction between immune checkpoint proteins and their ligands to prevent T cell inactivation (10, 11). However, not all HCC patients respond to immunotherapy. Moreover, monotherapy has a lower objective response rate (ORR) and no significant improvement in OS (12, 13). Therefore, researchers are exploring more effective combination therapies for HCC. Recently, the combination of ICIs and antiangiogenic drugs has shown promising results (14). More studies are also exploring the use of different types of ICIs in combination with various targeted drugs. In this review, we provide the latest advances in the use of ICIs combined with targeted drugs (targeted-immune combination) and targeted-immune combination other regimens for the treatment of aHCC or unresectable HCC (uHCC). Additionally, we provide an outlook on future prospects and potential developments in this evolving therapeutic landscape.

## 2 Immune checkpoint inhibitors combined with targeted drugs

**Figure 2:**
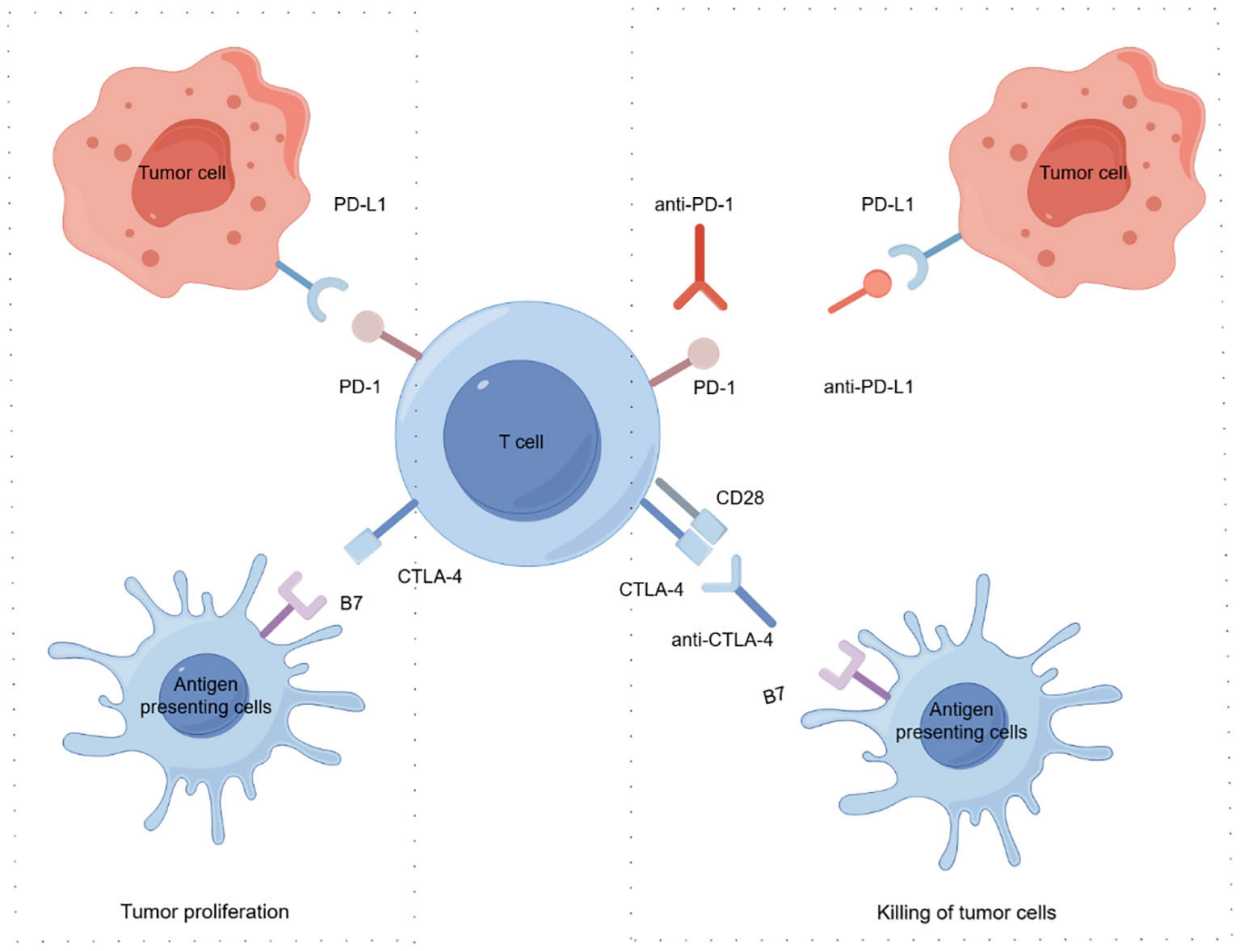
Mechanisms of tumor immune evasion and suppression of immune checkpoints following restoration of anti-tumor immunity Tumor cells evade immune surveillance by promoting immune checkpoint activation. Tumor cells express the immune checkpoint activator PD-L1 and produce antigens, which are captured by antigen presenting cells. These cells present antigens to cytotoxic CD8^+^ T cells through the interaction of major histocompatibility complex (MHC) molecules and T cell receptor (TCR). T cell activation requires costimulatory signaling mediated by B7 and CD28 interactions. Inhibitory signals from CTLA-4 and PD-1 checkpoints inhibit T cell responses and promote tumor proliferation. ICIs, such as anti-PD-L1, anti-PD-1, and anti-CTLA-4, block immunosuppressive checkpoints (CTLA-4, PD-1, and PD-L1, respectively), thereby restoring anti-tumor immune responses. By Figdraw.

Currently, anti-PD-L1 includes atezolizumab and durvalumab and so on (15). Anti-PD-1 mainly includes nivolumab, pembrolizumab, sintilimab, camrelizumab, tislelizumab and so on (16). Anti-cytotoxic T lymphocyte antigen-4 (CTLA-4) includes tremelimumab and ipilimumab and so on (17). The clinical application of ICIs represents a revolutionary milestone in oncology, but ICIs has a low response rate. Increasingly, clinical studies are combining ICIs with other treatments to achieve better treatment results and improve patient survival (**Figure 2**) (18). Sorafenib was the first oral tyrosine kinase inhibitor (TKI) approved for the treatment of advanced HCC (19). Subsequently, other TKIs such as lenvatinib, regorafenib, cabozantinib, and vascular endothelial growth factor receptor (VEGFR) inhibitors like ramucirumab and VEGF inhibitors like bevacizumab have been approved as first- or second-line treatments (20–23). More recently, the combination of ICIs and VEGF inhibitors (atezolizumab plus bevacizumab) has been approved for the treatment of aHCC (19). Studies have demonstrated the efficacy and safety of anti-PD-1 combined anti-angiogenesis therapy in a real-world cohort of patients with uHCC (24). Additionally, anti-PD-1 combined with TKIs has proven to be an effective and safe strategy for patients with portal vein tumor thrombus (PVTT) (25). A Phase I/II study showed that BMS-986,205 combined with nivolumab showed a DCR of 50% and no incidence of grade 4-5 adverse events (AEs), suggesting that this combination offers manageable safety and lasting benefit in unresectable/metastatic HCC patients (26).

### 2.1 Anti-PD-L1 plus targeted drugs

#### 2.1.1 Atezolizumab plus bevacizumab

The combinations of atezolizumab and bevacizumab are summarized in **Table 1**.

**Table 1:** Clinical trials with atezolizumab and bevacizumab in HCC.

Combinations	Trial	patient Number	mOS	mPFS	ORR/DCR	3 or/and 4 AEs	Ref.
atezolizumab plus bevacizumab	phase 1b(GO30140)NCT02715531	A 104, F109	A:17.1 months; F:8.3 months	F:5.6 months	A:ORR:36%,DCR:71%	A:20%; F:8%	(27)
atezolizumab plus bevacizumab	phase III(IMbrave050)NCT04102098	668	NA	NA	NA	41%	(28)
atezolizumab plus bevacizumab	phase III(IMbrave150)NCT03434379	501	NA	6.8 months	ORR:28%	57%	(29)
atezolizumab plus bevacizumab	12 months after the primary analysis of IMbrave150	501	19.2 months	6.9 months	ORR:30%	43%	(30)
atezolizumab plus bevacizumab	German	100	20.3 months	6.3 months	ORR:36%	64%	(31)
atezolizumab plus bevacizumab	Taiwan	40	24.9 months	8.6 months	DCR:85%	3 AEs:67.5% or 4 AEs:50%	(32)
atezolizumab plus bevacizumab	Taiwan	35	22.2 months	5.2 months	ORR:23%,DCR:72%	9%	(33)
atezolizumab plus bevacizumab	Japan	52	NR	4.7 months	ORR:15.4%,DCR:57.7%	Any grade AEs:69%	(34)
atezolizumab plus bevacizumab	Korean	121	NR	6.5 months	ORR:24%,DCR:76%	10.70%	(35)
atezolizumab plus bevacizumab	older (age ≥ 65 years) and younger (age < 65 years)	191	older:14.9 months,younger:15.1 months	older:7.1 months,younger:5.5 months	ORR:older:27.6%,younger:20%;DCR:older:77.5%,younger:66.1%	older:20.7%;younger:20.0%	(36)
atezolizumab plus bevacizumab	elderly and non-elderly	317	3, 6, and 9 months:elderly:95.8%, 90.0%, 83.9%, non-elderly:96.2%, 89.5%,80.8%	3 and 6 months:elderly:76.6% and 50.3%;non-elderly:74.8% and 54.2%	ORR:elderly:30.5%,non-elderly:22.8%;DCR:elderly:83.9%,non-elderly:80.2%	≥3 AEs:elderly:39.2%;non-elderly:21%	(37)
atezolizumab plus bevacizumab	overweight (BMI ≥ 25) and non-overweight (BMI < 25)	191	overweight:15.1 months;non-overweight:14.9 months	overweight:7.1 months;non-overweight:6.1 months	ORR:overweight:27.2%;non-overweight:22.0%;DCR:overweight:74.1%;non-overweight:71.9%	≥3 AEs:overweight:19.2%;non-overweight:21.7%	(38)
atezolizumab plus bevacizumab	without PH and with PH	146	without PH:18.4 months; with PH:18.8 months	without PH:8.6 months;with PH:5.8 months	ORR:without PH:31.7%;with PH:26.8%	without PH:78%;with PH:79.9%	(39)

ICIs, immune checkpoints inhibitors; ORR, objective response rate; DCR, disease control rate; mPFS, median progression free survival; mOS, median overall survival; AEs, adverse events; NR, not reached; NA, not available.

A Phase 1b study has shown that atezolizumab combined with bevacizumab is effective and has a tolerable safety profile in uHCC patients who have not previously received systemic therapy (27). The ORR (36% in group A) and disease control rate (DCR) (71% in group A). Treatment with Atezolizumab plus bevacizumab in group F resulted in clinically meaningful improvement in median progression-free survival (mPFS) by 2.2 months and a reduced risk of progression or death (27). In the phase 3 IMbrave050 study, recurrence-free survival improved in patients who received atezolizumab plus bevacizumab compared to those under active surveillance after HCC resection or ablation (28). The IMbrave150 trial demonstrated that atezolizumab plus bevacizumab significantly improved median overall survival (mOS) and mPFS in uHCC patients compared to sorafenib after a median follow-up of 8.6 months (29). 12 months after initial analysis of IMbrave150, atezolizumab plus bevacizumab maintained consistent safety and tolerability (30). After an additional 12 months of follow-up, this combination achieved a mOS of 19.2 months, mPFS of 6.9 months, and ORR of 30% compared to sorafenib (30). Atezolizumab plus bevacizumab showed good efficacy and safety in patients with uHCC and partially advanced liver cirrhosis in a real-world setting (40). Among 171 patients (BCLC stage A:B:C:D=5:68:96:2), this combination effective as both first-line and post line therapy (41). In a German study, the combination significantly improved rates of OS and PFS (31). In Taiwan, the combination provided a 37.5% response rate in patients with aHCC who received systemic therapy for the first time, with a mPFS of 8.6 months and a mOS of 24.9 months (32). Patients who achieved an objective tumor response had a 24-month OS rate of 81%, while those with stable disease had a 24-month OS rate of 57% (32). The most common adverse events were proteinuria and hypertension (32). The Taiwan-Tainan Medical Oncology Group H01 Trial, involving 35 patients reported an overall response rate of 51%, ORR of 23%, and DCR of 72% (33). The mPFS and mOS were 5.2 months and 22.2 months, respectively (33). In Japan, patients receive atezolizumab plus bevacizumab as first line (n=23), second line (n=16), third line (n=6), fourth line (n=3), fifth line (n=3), or sixth line (n=1) (34). According to Response Evaluation Criteria in Solid Tumors (RECIST), the ORR and DCR for all patients were 15.4% and 57.7%, respectively (34). Patients who received the combination as first-line treatment were significantly longer than those who received atezolizumab as late-stage treatment (34). In Korean patients with aHCC, atezolizumab plus bevacizumab showed efficacy and safety consistent with the Phase III trial results. The ORR of 121 patients was 24.0%, DCR was 76%, and the mPFS was 6.5 months (35). Studies have also shown that patients of different ages, particularly the elderly, benefit from atezolizumab and bevacizumab (36, 37). This combination has proven effective in patients with HCC who are overweight, have portal hypertension (PH), and have relatively good liver function (38, 39, 42–44). Early changes in HCC perfusion could predict the long-term therapeutic response of atezolizumab plus bevacizumab, facilitating personalized treatment for HCC patients (45).In case analysis, the study has found that a patient with unresectable aHCC with major portal vein tumor thrombus (Vp4 PVTT) cases responded significantly to atezolizumab plus bevacizumab (46). This combination showed a powerful anti-tumor effect in such cases (46). In another case, patient with hepatocellular and cholangiocarcinoma (CHC) and multiple lymph node metastases obtained PFS of 7.5 months after treatment with atezolizumab plus bevacizumab (47). There was also a case of unresectable multinodular HCC with a complete tumor response following atezolizumab/bevacizumab treatment, leading to liver transplantation due to liver failure. This therapy resulted in complete pathological remission of aHCC, but the safety of long-term treatment needed further evaluated (48). A 49-year-old woman with primary large HCC complicated with portal vein tumor thrombosis responded favorably to atezolizumab in combination with bevacizumab after disease progression through pembrolizumab and Lenvatinib (49). This suggests that HCC patients who are resistant to anti-PD-1 might benefit from anti-PD-L1, providing a potentially promising strategy for the treatment of HCC (49).

The combinations of ICIs, targeted drugs, and other treatments are summarized in **Table 2**.

**Table 2:** Clinical trials with ICIs and targeted drug and others treatments in HCC.

Combinations	Trial	patient Number	OS	PFS	ORR/DCR	3 or/and 4 AEs	Ref.
avelumab plus axitinib	Phase 1b(NCT03289533)	22	14.1 months	5.5 months	ORR:13.6%,DCR:68.2%	3 Aes:72.7%	(50)
atezolizumab plus cabozantinib	phase III(COSMIC-312)	837	16.5 months	6.9 months	NA	66%	(51)
atezolizumab plus bevacizumab plus lenvatinib	retrospective clinical study	25	10.5 months	6.0 months	ORR:25.0%,DCR:95.0%	30%	(52)
anti-PD-1 plus lenvatinib	retrospective clinical study	118	18.4 months	10.6 months	ORR:32.7%,DCR:80.0%	63.60%	(53)
anti-PD-1 plus lenvatinib	phase II	124	23.9 months	8.9 months	ORR:53.6%	42.90%	(54)
anti-PD-1 plus lenvatinib	real-world report	87	22.9 months	7.5 months	ORR:45.0%	42.50%	(55)
anti-PD-1 plus lenvatinib	phase III(NCT03713593)	1309	21.2 months	8.2 months	NA	62%	(56)
anti-PD-1 plus lenvatinib	retrospective clinical study	71	NA	9.3 months	ORR:34.1%,DCR:84.1%	NA	(57)
anti-PD-1 plus lenvatinib	retrospective clinical study	159	21.7 months	11.3 months	ORR:38.9%,DCR:92.6%	5.56%	(58)
anti-PD-1 plus lenvatinib	phase Ib	116	22 months	9.3 months	ORR:46%	64%	(59)
anti-PD-1 plus sorafenib	retrospective clinical study	93	19.23 months	8.63 months	ORR:21.4%,DCR:83.9%	32.10%	(60)
anti-PD-1 plus apatinib	phase Ib/II	28	13.2 months	3.7 months	ORR:10.7%	≥3 Aes:92.9%	(61)
anti-PD-1 plus rivoceranib	phase III(CARES-310)	842	22.1 months	5.6 months	ORR:25%	81%	(62)
anti-PD-1 and regorafenib	retrospective clinical study	17	NR	5.09 months	ORR:41.2%,DCR:64.7%	17.64%	(63)
Targeted-immune combination TACE	retrospective clinical study	139	14 months	10 months	ORR:38.7%,DCR:69.4%	3 Aes:3.2%	(64)
Targeted-immune combination TACE	retrospective clinical study	65	26.8 months	11.7 months	ORR:44.4%,DCR:93.3%	73.10%	(65)
Targeted-immune combination TACE	retrospective clinical study	168	29 months	16.2 months	ORR:76.7%,DCR:96.7%	≥3 Aes:30%	(66)
Targeted-immune combination TACE	retrospective clinical study	204	NR	24.1 months	ORR:70.4%,DCR:100.0%	35.30%	(67)
Targeted-immune combination TACE	retrospective clinical study	234	21.7 months	6.3 months	ORR:41.25%,DCR:86.25%	22.50%	(68)
Targeted-immune combination TACE	retrospective clinical study	84	26.7 months	8.2 months	ORR:86.96%,DCR:100%	≥3 Aes:56.53%	(69)
Targeted-immune combination TACE	retrospective clinical study	152	20.5 months	10.2 months	ORR:54.3%	≥3 Aes:43.6%	(70)
Targeted-immune combination TACE	retrospective clinical study	53	NA	8.5 months	ORR:54.9%,DCR:84.3%	≥3 Aes:32.1%	(71)
Targeted-immune combination TACE	retrospective clinical study	92	16.9 months	7.3 months	ORR:56.1%,DCR:85.4%	3 Aes:36.7%	(72)
Targeted-immune combination TACE	retrospective clinical study	41	21.7 months	14.5 months	ORR:68.3%	17.00%	(73)
Targeted-immune combination TACE	retrospective clinical study	169	10.9 months	19.6 months	ORR:66.7%,DCR:82.6%	14.80%	(74)
Targeted-immune combination TACE	retrospective clinical study	246	19.5 months	9.7 months	ORR:73%,DCR:89%	34.70%	(75)
Targeted-immune combination TACE	retrospective clinical study	87	24 months	9.7 months	ORR:52.4%,DCR:83.3%	19.00%	(76)
Targeted-immune combination HAIC	phase I(NCT04191889)	40	NR	10.38 months	ORR:77.1%,DCR:97.1%	≥3 Aes:74.3%	(77)
Targeted-immune combination HAIC	retrospective clinical study	405	18.0 months	10.0 months	DCR:83%	0.00%	(78)
Targeted-immune combination HAIC	retrospective clinical study	142	26.3 months	11.5 months	ORR:61.8%	89.80%	(79)
Targeted-immune combination HAIC	retrospective clinical study	248	17.7 months	10.9 month	ORR:59.5%	≥3 Aes:4.76%	(80)
Targeted-immune combination HAIC	retrospective clinical study	27	NR	10.6 months	ORR:63.0,DCR:92.6%	3 Aes:55.6%	(81)
Targeted-immune combination HAIC	retrospective clinical study	210	14.6 months	8.37 months	ORR:60.6%,DCR:84.8%	28.70%	(82)
Targeted-immune combination HAIC	retrospective clinical study	123	482 days	208 days	ORR:59%	33%	(83)
Targeted-immune combination radiotherapy	retrospective clinical study	33	9.8 months	8.0 months	ORR:76.6%	27%	(84)
Targeted-immune combination radiotherapy	retrospective clinical study	202	15.8 months	8.3 months	ORR:89.5%,DCR:94.7%	5.30%	(85)
Targeted-immune combination chemotherapy	retrospective clinical study	65	NR	10.6 months	ORR:67.3%	59.10%	(86)
Targeted-immune combination chemotherapy	retrospective clinical study	104	14.3 months	8.63 months	ORR:52.8%	41.50%	(87)
Targeted-immune combination chemotherapy	phase II(NCT04411706)	47	NA	9.0 months	ORR:50%,DCR:91.3%	28.30%	(88)
Dual immune checkpoint inhibitors combined with targeted drugs	phase I/II(CheckMate 040)	98	22.1	4.3 months	ORR:29%	74%	(89)

ICIs, immune checkpoints inhibitors; ORR, objective response rate; DCR, disease control rate; mPFS, median progression free survival; mOS, median overall survival; AEs, adverse events; NR, not reached; NA, not available.

#### 2.1.2 Avelumab plus axitinib

A phase 1b study enrolled 22 Japanese patients who were treated with a combination of avelumab plus axitinib (50). The minimum follow-up time was 18 months. Grade 3 treatment-related adverse events (TRAEs) occurred in 16 patients (72.7%) (50). No grade 4 TRAEs or treatment-related deaths were reported (50).

#### 2.1.3 Atezolizumab plus cabozantinib

The COSMIC 312 trial showed that atezolizumab plus cabozantinib had a PFS benefit compared to sorafenib in the first 372 randomized patients (90). However, there was no difference in OS in the interim analysis among the intention-to-treat population (90). In the most recent analysis, cabozantinib did not show an OS benefit compared to sorafenib in the intent to treat population (51). Nevertheless, subgroup analyses showed that potential benefits of cabozantinib in patients with hepatitis B etiology and baseline AFP of 400 ng/mL or higher. The PFS benefit of combination therapy was maintained with longer follow-up and in a larger group of intended treaters (51).

#### 2.1.4 Atezolizumab plus bevacizumab plus lenvatinib

Although lenvatinib therapy did not provide a pseudo-combined immunotherapy effect after atezolizumab plus bevacizumab failure, it may still be comparable as a second-line treatment (91). Patients treated with atezolizumab and bevacizumab after lenvatinib treatment may experience rapid tumor growth and subsequent shrinkage (92). Lenvatinib has been effective and safe for treating aHCC patients who were previously treated with atezolizumab plus bevacizumab. It can effectively control anorexia, general fatigue and other adverse reactions without compromising its therapeutic effect (52). In a 68-year-old uHCC patient with adrenal metastases, lenvatinib was effective after atezolizumab plus bevacizumab treatment failure (93).

### 2.2 Anti-PD-1 plus targeted drugs

Anti-PD-1 improved OS and PFS in patients with aHCC (94). The efficacy of anti-PD-1 therapy, whether used alone or in combination with TKIs, varies depending on the metastatic site. Notably, a high response rate in vascular metastasis was associated with longer PFS (94). Anti-PD-1 might provide a synergistic benefit when used in conjunction with conventional therapy, potentially enhancing vascular responses in other organs (94).

#### 2.2.1 Anti-PD-1 plus lenvatinib

The study also showed that the ORR (32.7%), DCR (80.0%), mPFS (10.6 months) and mOS (18.4 months) in combination with anti-PD-1 and lenvatinib were significantly higher than those in anti-PD-1 group (53). The simultaneous use of anti-PD-1 and lenvatinib could significantly improve the clinical outcome of aHCC (95). Different anti-PD-1 combined with lenvatinib have shown a good safety profile, guiding treatment options in patients with uHCC (96). Anti-PD-1 plus lenvatinib was a safe and effective conversion therapy for unresectable patients with aHCC (54). This combination was a promising new strategy for the treatment of HCC patients (97). Anti-PD-1 and lenvatinib therapy demonstrated an ORR of 45.0%, a PFS of 7.5 months and an OS of 22.9 months. These data suggested that Lenvatinib combined with nivolumab was a potential combination for aHCC (55). In first-line therapy for patients with aHCC, the LEAP-002 study showed that the pembrolizumab plus lenvatinib had an OS of 21.2 months and a PFS of 8.2 months (56). The most common grade 3-4 TRAEs were hypertension (56). Clinical data for pembrolizumab plus lenvatinib showed no unexpected adverse effects, showing positive responses and survival rates even in patients with high-risk tumors and Child-Pugh B status (57). In lenvatinib plus sintilimab group, the mOS was 21.7 months, and mPFS was 11.3 months (58). According to the mRECIST criteria, the ORR was 38.9%, and the DCR was 92.6% (58). ICIs plus lenvatinib provided significantly higher OS and PFS than lenvatinib (98). In addition, ICIs plus lenvatinib had significantly higher ORR (41.5%) and DCR (72.3% vs 46.7%) per RECIST v1.1 than lenvatinib (98). In a phase Ib single-arm study showed that lenvatinib plus pembrolizumab had a longer mPFS of 9.3 months (by mRECIST; 8.6 months by RECIST v1.1) per IIR, and mOS of 22.0 months (59). A 63-year-old male patient received combination immunotherapy with Lenvatinib and Pembrolizumab. He had a complete response (CR) nine months after treatment (99). Now, 22 months since initial treatment, there was no clinical evidence of disease progression. The current OS was 22 months (99).

#### 2.2.2 Anti-PD-1 plus sorafenib

In hepatitis virus-associated HCC, both the mOS (19.23 months) and mPFS (8.63 months) were significantly improved in the TKIs (sorafenib or lenvatinib or regorafenib) plus ICIs (camrelizumab or sintilimab) group compared to the TKIs group (60). The DCR was also significantly higher in the TKI-ICIs group at 83.9% (60). Compared with anti-PD-1 alone, the combination of anti-PD-1 (nivolumab or pembrolizumab) and sorafenib showed better tumor control with an ORR of 22.4%, longer PFS (3.87 months) and OS (100). Importantly, there was no significant increase in grade 3 or 4 AEs, and a significant reduction in AFP levels was observed (100). Anti-PD-1 (nivolumab or pembrolizumab) therapy increases CD4^+^ and CD8^+^ T cell infiltration and provides vascular protection, which is beneficial for subsequent multi-kinase inhibitor therapy. In this sequence, sorafenib acts as an immune stimulator by promoting CD8^+^ T cell infiltration (101). A 62-year-old man showed extensive tumor reduction after multiple treatments sintilimab combined with sorafenib. This suggested that the protocol was a promising therapeutic strategy for the treatment of HCC (102).

#### 2.2.3 Anti-PD-1 plus cabozantinib

A 71-year-old metastatic HCC patient with RET amplification, high tumor mutation burden, and positive PD-L1 expression responded well to the combination of cabozantinib and nivolumab, achieving a PFS of over 25 months (103). Cabozantinib and nivolumab may be a good option for patients with aHCC, especially those with bone metastases (103). Studies have shown that TKIs (lenvatinib or apatinib) plus anti-PD-1 (nivolumab or pembrolizumab or sintilimab) is safe and effective in the treatment of uHCC (104). The mOS was 27.0 months and the 1-year OS rate was 83.6%. The mPFS was 15.0 months and the 1-year PFS rate was 77.0% (104).

#### 2.2.4 Anti-PD-1 plus apatinib

In patients with advanced primary liver cancer (PLC), camrelizumab and apatinib achieved a manageable safety profile and good efficacy. The mPFS and mOS were 3.7 months and 13.2 months, respectively (61). A 250mg dose of apatinib is recommended as a combination therapy for further study of late-stage PLC therapy (61).

#### 2.2.5 Anti-PD-1 plus rivoceranib

A Phase 3 study has shown that camrelizumab-rivoceranib met both primary endpoints, with an improvement of 6.9 months in mOS and 1.9 months in mPFS (per RECIST 1.1 by the BIRC) compared to the sorafenib group (62). The risk of death was reduced by 38% and the risk of progression or death by 48% (62). The combination therapy resulted in significantly higher response rates, longer lasting responses and higher DCR compared to the sorafenib group (62).

#### 2.2.6 Anti-PD-1 plus regorafenib

Regorafenib combined with anti-PD-1 (camrelizumab or sintilimab) was safe and effective for treating aHCC, with a low incidence of severe AEs (63). Seventeen patients with BCLC-B and BCLC-C HCC were followed up for a median of 7.62 months (63). The ORR and DCR were 41.2% and 64.7%, respectively, and the mPFS was 5.09 months (63). In a refractory patient previously treated with sorafenib, progressive disease occurred during treatment with anti-PD-1 (nivolumab) and the anti-GITR (BMS-986156) in a Phase 1 clinical trial (105). Subsequently, a prolonged tumor response was achieved during third-line therapy with regorafenib according to RECIST v.1.1 criteria (105).

## 3 Targeted-immune combination local therapy

Based on targeted-immune combination, combined with local treatment means such as intervention and radiotherapy, the comprehensive treatment can improve the treatment efficiency of middle HCC and aHCC.

### 3.1 Targeted-immune combination TACE

The mOS of 14 months, mPFS of 10 months and ORR of 38.7% in the treatment of aHCC patients with TACE combined with atezolizumab and bevacizumab were significantly improved, with acceptable safety (64). This combination was effective reducing the early recurrence of HCC without severe complications (106). In a 74-year-old patient with HCC, the liver tumor achieved complete remission after TACE, but lung, bone, and lymph node metastases were observed (107). These metastases eventually decreased, showing partial response after continuous administration of atezolizumab plus bevacizumab (107). Compared to TACE combined with sorafenib, TACE combined with sorafenib and ICIs (camrelizumab or sintilimab) was a potentially safe and effective treatment option for patients with aHCC who have previously received local regional therapy. These patients had higher DCR (82.8%), longer mPFS (6.9 months), and longer mOS (12.3 months) (108). TACE combined with lenvatinib plus anti-PD-1 (TACE-L-P) provided better treatment response and survival benefits, with manageable adverse events (65–70, 109). In 51 evaluable patients, the confirmed ORR was 54.9% and the DCR was 84.3% (71). The mPFS was 8.50 months (71). Grade ≥3 TRAEs was developed in 32.1% of patients (71). No new safety signals detected (71). TACE-L-P (camrelizumab or sintilimab) might have good anti-tumor activity in the treatment of uHCC. Toxicity was manageable, no unexpected safety signals (71). In HCC patients with portal vein tumor thrombus (PVTT), the DCR (80.00%), ORR (38.57%), mOS (23.5 months) and mPFS (7.5 months) of TACE-L-P (pembrolizumab or sintilimab) were significantly better than those of TACE+lenvatinib (110). The patients in TACE-L-P (sintilimab or tislelizumab or camrelizumab) group had prolonged mOS (16.9 months), longer mPFS (7.3 months) and higher ORR (56.1%) and DCR (85.4%) than those in TACE-L group (72). TACE-L-P (camrelizumab or tislelizumab or sintilimab) combined with Vp4 was effective and tolerated in treating uHCC, with a high tumor response rate and good prognosis (73). For HCC PVTT patients, compared with TACE combined with apatinib alone (TACE-A), TACE combined with apatinib and anti-PD-1 (TACE-A-P) significantly improved PFS, OS, and ORR, and the TRAEs was safe and controllable (111). TACE plus apatinib and TACE plus apatinib plus camrelizumab were feasible in patients with uHCC with a manageable safety profile. TACE plus apatinib plus camrelizumab showed additional benefits compared to TACE plus apatinib (112). The TACE plus donafenib plus toripalimab group showed higher ORR (66.7%) and DCR (82.6%), longer mPFS (10.9 months) and longer mOS (19.6 months) compared to the TACE plus sorafenib group (74). Patients treated with TACE combined with TKIs and ICIs (nivolumab or pembrolizumab or camrelizumab) had significantly longer OS than those treated with TKIs plus ICIs without TACE. Both groups tolerated severe AEs well, with no significant difference in incidence (75). Compared with TACE combined with molecularly targeted agents (sorafenib or lenvatinib or apatinib or regorafenib or bevacizumab), TACE combined with molecularly targeted agents plus ICIs (camrelizumab or sintilimab or pembrolizumab or tislelizumab or atezolizumab) improved the survival and tumor response of uHCC patients, and the toxicity is controllable (76). The mOS (24.00 months) and mPFS (9.70 months) were both significantly longer (76). HCC patients treated with TACE combined with molecular targeted agents (sorafenib or lenvatinib or apatinib or regorafenib) plus ICIs (camrelizumab), the formation of liquefaction necrosis increased (113). Larger tumor size and higher AFP levels were associated with more liquefaction necrosis in the tumor (113).

### 3.2 Targeted-immune combination HAIC

One study (NCT04191889) evaluated the benefit of camrelizumab and apatinib combined with HAIC-FOLFOX in patients with BCLC-C HCC (77). Thirty-five patients were enrolled. The ORR was 77.1% and the DCR was 97.1% (77). The mPFS was 10.38 months (77). The most common treatment-related AEs with grade ≥3 or above included reduced lymphocyte count (37.1%) and reduced neutrophil count (34.3%) (77). This combination showed encouraging results and manageable safety concerns (77). The HAIC plus anti-PD-1 group had a longer mOS of 18.0 months and a longer mPFS of 10.0 months, as well as a higher DCR (83%) and intrahepatic response (85%) (78). HAIC-FOLFOX plus lenvatinib plus anti-PD-1 (pembrolizumab or sintilimab or toripalimab or camrelizumab or tislelizumab) was an effective and safe treatment for HCC patients with PVTT. There were significant improvements in OS (26.3 months), PFS (11.5 months) and ORR (61.8%) (79). Pembrolizumab plus lenvatinib and HAIC prolonged median PFS (10.9 months) and OS (17.7 months) in newly treated uHCC patients with PD-L1 staining (80). The mOS was 43.6 months and post progression‐free survival (PPS) was 35.6 months in anti-PD‐1 plus lenvatinib plus HAIC group (114). Anti-PD-1 (camrelizumab or sintilimab ot toripalimab or nivolumab) combined with TKIs (lenvatinib or sorafenib or regorafenib or apatinib) and HAIC was safe and effective for aHCC. The ORR was 63.0%, the DCR was 92.6%, and the median PFS was 10.6 months. The most common grade 3 AEs were pain (7.4%) and elevated ALT (7.7%) (81). A meta-analysis has shown that HAIC based therapy improved the prognosis of patients with HAIC (115). Although HAIC combined with anti-PD-1/anti-PD-L1 (triple therapy) increased the incidence and severity of AEs, it produced higher ORR, longer PFS and OS compared to angiogenesis inhibitors plus anti-PD-1/anti-PD-L1 (115). Initial hepatic artery intervention plus anti-PD-1 and targeted therapy led to longer median PFS (8.37 months) and OS (up to 14.6 months) in BCLC-C HCC patients (82). Transarterial interventional therapy combined with TKIs (lenvatinib or sorafenib or apatinib) and anti-PD-1 (triplet regimen) produced excellent results and controllable AEs in patients with HCC and severe PVTT. Compared to double regimens, the triplet regimen resulted in longer median PFS (208 days) and OS (482 days) (83). Skeletal muscle index (SMI) combined with interventional therapy with ICIs (toripalimab or camrelizumab) and TKIs (lenvatinib) highlighted sarcopenia as an independent risk factor for OS in HCC patients treated with sorafenib or regorafenib, which could be of great help for personalized medical treatment of HCC patients (116). The meta-analysis suggested that triple therapy with TACE/HAIC, TKIs, and ICls provided clinical benefit for uHCC in both short and long-term outcomes without an increase in severe AEs, though further validation is needed (117).

### 3.3 Targeted-immune combination radiotherapy

In comparison to the combination of ICIs (pembrolizumab or camrelizumab or sintilimab or atezolizumab) and antiangiogenic therapy (lenvatinib or sorafenib or donafenib or bevacizumab or apatinib), the inclusion of RT has improved DCR and survival outcomes in aHCC patients (118). The safety profile of this triple therapy was satisfactory (118). For HCC patients, transarterial radioembolization (TARE) using Y-90 resin microspheres showed similar results to atezolizumab-bevacizumab (AB) (119). The mOS was 15.0 and 14.9 months for TARE and AB, respectively (119). The mPFS was 4.4 and 6.8 months for TARE and AB, respectively (119). ORR were 19.8% and 25% with TARE and AB, respectively (119). Atezolizumab plus bevacizumab combined with TARE improved OS and PFS outcomes compared to TARE alone (120). In a cohort of 30 patients with PLC and extrahepatic portal vein tumor thrombus (ePVTT), combining intensity-modulated radiotherapy (IMRT) with systemic atezolizumab systemic atezolizumab and bevacizumab yielded an ORR of 76.6%. The median OS was 9.8 months, and the median PFS was 8.0 months (84). Patients with aHCC treated with radiotherapy before and/or during nivolumab therapy had significantly higher PFS and OS, with generally acceptable toxicity profiles (121). In HCC patients, PVTT was more sensitive to radiotherapy (RT) than primary tumor (PT). Combining RT with anti-angiogenesis and ICIs in aHCC patients created surgical opportunities and may be promising for low-stage HCC patients with PVTT (122). Proton beam radiotherapy (PBT) combined with anti-PD-1/anti-PD-L1 was safe, with no accidental AEs. Concurrent therapy effectively treated aHCC through sustained local tumor necrosis and effective systemic tumor control (123). The mOS for the entire cohort was 12.9 months. In patients with advanced uHCC, immunotherapy with Y90-RE or nivolumab or atezolizumab/bevacizumab within 90 days appeared to be well tolerated, with a low incidence of severe AEs (124). Sequential ICIs (anti-PD-1: sintilimab or camrelizumab, ati-PD-L1: atezolizumab) plus bevacizumab plus bevacizumab therapy after radiotherapy for PVTT in patients with HCC was safe and feasible, potentially prolonging PFS (125). HCC patients treated with Y90+ICI had better ORR (89.5%) and DCR (94.7%) than those treated with Y90 plus TKI (85). The mPFS was 8.3 months and mOS was 15.8 months, patients had no significant combination therapy AEs attributed to radioembolization (85).

### 3.4 Targeted-immune combination chemotherapy

Atezolizumab plus bevacizumab combined with oxaliplatin (HAIC-FOLFOX) showed ORR of 67.3% based on mRECIST criteria and 44.2% based on RECIST 1.1 criteria (86). The mPFS of patients was 10.6 months (86). AEs were controllable, suggesting this combination may be a potential treatment option for aHCC (86). Anti-PD-1 (toripalimab) plus lenvatinib with Gemox chemotherapy as a first-line treatment option for advanced ICC (87). The mOS was 14.3 months and the mPFS was 8.63 months, and the median ORR was 52.8% (87). The incidence of grade 3 and 4 AEs was 41.5%, which was acceptable, tolerable and controllable (87). A single-arm Phase II clinical study met its pre-set primary endpoint, showing that sintilimab combined with apatinib plus capecitabine had a good safety profile and antitumor activity as a first-line treatment for uHCC (88). The ORR based on blinded independent image evaluation was 50.0% and the DCR was 91.3% (88).

### 3.5 Targeted-immune combination ablation

A 38-year-old male patient received prophylactic TACE after surgery (126). Three months after surgery, the patient developed multiple liver metastases (126). He underwent atezolizumab and bevacizumab combined with intratumor cryoablation (126). After treatment, the patient’s tumor exhibited extensive necrosis, the disease has been effectively controlled (126).

## 4 Dual immune checkpoint inhibitors combined with targeted drugs

Tumor cells evade the immune system in several ways, so combining ICIs with different mechanisms of action could be an interesting therapeutic strategy (127). Inhibition of the B7-CTLA-4 pathway by anti-CTLA-4 play an anticancer role by increasing the level of activated CD8^+^ T cells in the lymph nodes (128).

A meta-analysis showed that combining anti-PD-1 with anti-PD-L1 for uHCC improved OS, PFS, ORR, DCR, especially in patients with HBV infection and among Asian populations (129). While the incidence of any grade and grade 3-5 TRAEs was higher with combination therapy, the safety was manageable (129). Another Meta-analysis showed that anti-PD-1/anti-PD-L1 was superior to sorafenib and placebo in OS, PFS, ORR and DCR in uHCC patients, especially when anti-PD-L1 was combined with anti-VEGF (130). However, the incidence of AEs was slightly higher in patients treated with anti-PD-1/anti-PD-L1 (130). In cohort 6 of the CheckMate 040 study, the ORR for nivolumab and cabozantinib was 17%, and for the triplet therapy (nivolumab, ipilimumab, and cabozantinib) was 29% (89). The mPFS was 5.1 months and 4.3 months, and the mOS was 20.2 months and 22.1 months, respectively (89). The incidence of grade 3-4 TRAEs was 50% for the doublet and 74% for the triplet, with TRAEs leading to discontinuation in 11% and 23% of patients, respectively (89). Notreatment-related deaths occurred in either group (89). In a randomized Phase 1 trial, stereotactic body radiation therapy (SBRT) of nivolumab plus ipilimumab outperformed immunotherapy alone in patients with aHCC or uHCC (131). Adding 1mg/kg ipilimumab to the atezolizumab plus bevacizumab combination during induction was safe, showed acceptable toxicity and increased ORR and subsequently improved patient outcomes (132). For aHCC patients, the sequence of TKIs and ICIs treatment might not matter. Patients who are frail or have comorbidities that preclude them from tolerating the combination therapy (ICls and TKIs/anti-VEGF) might benefit from continuous exposure to both drug classes (133).

## 5 Challenges in combination therapy for HCC

However, challenges remain, including drug resistance and AEs in combination therapy. First, ICIs in combination with targeted drugs is unlikely to be cost-effective (134–137). Secondly, ICIs can encounter resistant (primary or acquired), which remains the leading cause of treatment failure (138). Drug resistance is complex and dynamic, as abnormal behavior at any step can lead to resistance. Therefore, developing new methods to reduce drug resistance is critical.

In addition, after ICIs treatment, an over-activated immune system can lose self-tolerance, leading to non-tumor auto-immune response, resulting in immune-related AEs (irAEs) (139). These effects are usually mild and manageable but can sometimes be life-threatening. Rash and itching are the most common clinical features (140). Other common adverse events include diarrhea and colitis, hepatotoxicity and elevated AST, elevated alkaline phosphatase and elevated ALT, thyroid dysfunction, lung, blood, and HBV reactivation (141–148). Therefore, necessary baseline assessment and screening should be performed before targeted-immune combination. For patients receiving immunotherapy, it is crucial to conduct routine medical history inquiry, manage underlying diseases, complete baseline screening, and adequate address underlying diseases or comorbidities before initiating immunotherapy. Baseline viral DNA screening and routine antiviral therapy for HBV patients.

## 6 Potential biomarkers of combination therapy

The combination of anti-PD-1/anti-PD-L1 plus anti-VEGF drugs may have significantly better clinical benefits (149, 150). However, not all HCC patients receiving combination therapy achieve the expected efficacy, and biomarkers are essential for predicting and evaluating treatment effect. A meta-analysis showed that atezolizumab in combination with bevacizumab was effective and well tolerated in treating aHCC (151). This combination demonstrated better tumor response rates in long-term, first-line, and low-dose therapy (151). Atezolizumab plus bevacizumab treatment could be expected to elicit an effective immune response in untreated uHCC patients (152–154). A low pretreatment neutrophil-to-lymphocyte ratio (NLR ≤ 2.22) might indicate longer OS (25.8 months) and PFS (14.0 months) for patients with uHCC treated with TACE plus TKIs (sorafenib or lenvatinib or apatinib) plus ICIs (camrelizumab) (155). Lenvatinib plus anti-PD-1 plays a unique immunomodulatory role by activating the immune pathway, reducing Treg cell infiltration, and inhibiting TGF-β pathway. Although these HCC do not respond to a single drug, they could benefit from the proposed combination drug (156). In mouse models, cabozantinib, especially when combined with anti-PD-1 therapy, induced neutrophil infiltration, reduced the immunosuppressive environment and enhanced antitumor activity compared with monotherapy. Patients with reduced active neutrophil phenotypes in their tumors (about 30% of cases) might benefit the most from this combination (157). Cryo-thermal ablation could transform HCC from a “cold” tumor to a “hot” tumor. This technique, combined with anti-PD-1 and anti-CTLA-4, might be a promising method for improving HCC prognosis (158). Anti-PD-1 therapy enhanced the anti-tumor immune response in liver cancer models. When used with sorafenib, this immunotherapy approach was effective only when simultaneously targeting the hypoxic and immunosuppressive microenvironment with drugs such as CXCR4 inhibitors (159). The study has shown that albumin-bilirubin (ALBI) grading and sorafenib treatment history are predictors of OS in HCC patients treated with lenvatinib. For patients with prior sorafenib experience, ICIs combined with lenvatinib achieved better OS than lenvatinib alone (160). Alpha-fetoprotein (AFP) is a potential alternative biomarker for atezolizumab plus bevacizumab in HCC (161). A 3-week AFP ratio of 1.4 or higher may predict refractory atezolizumab combined with bevacizumab (162). A reduction of ≥20% in AFP at 3 weeks was associated with longer OS and PFS, showing potential as a biomarker of response (163). AFP response was a predictor of disease control, PFS, and OS, making it useful for predicting treatment outcomes in uHCC patients receiving ICls (or not receiving TKIs or local therapy) (164). HCC with different genes can be divided into hot tumors and cold tumors based on tumor infiltrating CD8^+^ T cells in mice. Hot tumors respond to anti-PD-1 therapy, while cold tumors are more suitable for combination therapy with anti-PD-1 and sorafenib (165). Therefore, developing predictive biomarkers with high specificity and sensitivity is crucial to accurately identify HCC patients most likely to benefit from combination therapy.

## 7 Conclusions

More than 70% of HCC patients are diagnosed at intermediate to advanced stage (BCLC stage B, C, or D) and require systemic treatment. Traditional TKI drugs, such as sorafenib, lenvatinib, have provided some hope, but their clinical efficacy is still unsatisfactory (166). Consequently, new strategies are being developed. ICIs have ushered a new era in the treatment of aHCC. The combination of ICIs and anti-VEGFA, represented by anti-PD-1/PD-L1 and anti-CTLA-4, provides patients with up to 35% more ORRs and is better tolerated than other approved treatments (40, 167). The approval of atezolizumab combined with bevacizumab establishes a new benchmark for the treatment of advanced HCC, with a mOS duration of 20 months. This raises the question of whether patients who benefit from atezolizumab combined with bevacizumab might benefit from targeted or targeted combination with other treatments. The studies analyzed in this review provide some evidence that each targeted drug and ICIs has unique immunomodulatory effects, and that the target population benefiting from these treatments may differ significantly. In addition, we describe the relevance of etiological dependent mechanisms that may influence the outcome of ICIs and their combinations. Effectively utilizing the synergistic effect of different anti-tumor mechanisms will be the focus of future research and is expected to transform the current landscape of HCC treatment.

However, challenges remain, including drug resistance, predictive biomarkers of treatment effectiveness, and AEs in combination therapy. The potential causes of immune resistance to ICIs in HCC patients are complex and varied. These include the upregulation of immune checkpoints, impaired antigen recognition and presentation by immune cells, abnormal activation and proliferation of immunosuppressive cells, increased inhibitory cytokines, and the compromised proliferation and function of anti-tumor immune cells within the complex tumor microenvironment (TME) (168). Additionally, loss of tumor antigen expression, tumor heterogeneity, and dysbiosis of the gut microbiota are associated with ICI resistance (169). Combination therapy has become a primary treatment approach. Current combination therapies include ICIs with another ICI, ICIs with targeted therapy, chemotherapy, radiotherapy, traditional Chinese medicine, or modulation of the gut microbiota. The application of these treatments has not only improved the ORR of patients but also mitigated ICI resistance in HCC patients (170). In addition, ICIs lead to irAEs (140). Other common adverse events include diarrhea and colitis, hepatotoxicity and elevated AST, elevated alkaline phosphatase and elevated ALT, thyroid dysfunction, lung, blood, and HBV reactivation (141–148). Therefore, necessary baseline assessment and screening should be performed before targeted-immune combination.

At the same time, the treatment process should be closely monitored to detect and deal with adverse reactions promptly. Although ICIs are promising for HCC treatment, their ORR remains relatively low. The discovery and application of biomarkers for ICIs treatment effect will help clinicians effectively screen patients who benefit from ICIs treatment and make personalized treatment more precise. However, the biomarkers of ICIs beneficiaries of liver cancer are still in the exploratory stage or lack of strong evidence, and the combination of multiple biomarkers may be a new development trend. In the future, developing new immunosuppressants, exploring new therapeutic approaches, and discovering new prognostic biomarkers will be essential to achieving better therapeutic effects. More trials with larger sample sizes are needed to further validate the efficacy of ICIs and targeted-immune combination therapy for aHCC.

Current clinical studies show that a Phase I trial of nivolumab combined with cabozantinib as neoadjuvant therapy for three months resulted in 12 out of 15 patients successfully undergoing resection. Five patients achieving major pathological response. Several studies are exploring other preoperative combination regimens. Larger cohorts are needed to validate the role of ICIs in the adjuvant setting (171). Analysis of 58 specimens from patients who had residual tumor cells after preoperative TACE treatment revealed that TACE increased intratumoral inflammation and tumor antigen expression, thereby enhancing the efficacy of immunotherapy (172).

In addition, we should strengthen the study of immunotherapy for metastatic liver cancer, mixed liver cancer and NASH-associated liver cancer. HBV reactivation can occur in patients with HBV-associated HCC treated with ICIs. Routine monitoring of HBV DNA and effective prophylactic antiviral therapy are necessary before and during combination therapy. In addition, clinical trials of the immunotherapy combination regimen are ongoing, opening up numerous new possibilities for perioperative conversion therapy for advanced liver cancer. If preoperative and postoperative immunotherapy studies show positive results, perioperative survival for uHCC will improve, potentially making liver cancer clinically controllable If targeted-immune combination can transform initially unresectable patients with advanced liver cancer into resectable patients with survival benefits, then the treatment strategy and surgical indications will change, greatly improving survival outcomes.

However, current Chinese and foreign guidelines for the treatment of liver cancer exhibit several key differences (**Table 3**), reflecting distinct clinical practices, cultural preferences, and regional pharmaceutical approvals. Chinese guidelines, such as those from the Chinese Society of Clinical Oncology (CSCO), often recommend conventional therapies. This integration mirrors local clinical practices and cultural preferences. In contrast, foreign guidelines, including those from the American Association for the Study of Liver Diseases (AASLD) and the European Association for the Study of the Liver (EASL), primarily focus on evidence-based Western medical practices.

**Table 3:** Applicable conditions and guideline recommendations for different treatment methods in liver cancer.

Treatment Method	Applicable Conditions	Guideline Recommendations	Notes
Atezolizumab + Bevacizumab	First-line treatment, suitable for patients without severe bleeding risks	NCCN, AASLD, EASL	Increases ORR, provides conversion therapy opportunities for unresectable HCC
Nivolumab Monotherapy	First-line treatment, suitable for patients without severe liver dysfunction	NCCN	First inclusion in first-line treatment, immunotherapy monotherapy
Pembrolizumab	Second-line treatment, suitable for patients with prior treatment failure	NCCN, CSCO	Controversial as a second-line treatment
Nivolumab + Ipilimumab (O+Y)	Second-line treatment, suitable for patients with prior treatment failure	NCCN	Dual immunotherapy, first approved combination in second-line
Chemotherapy	First-line treatment, suitable for patients unable to receive immunotherapy or targeted therapy	Chinese guidelines	Not recommended by ESMO, highly recommended in China
Regorafenib	Second-line treatment, suitable for sorafenib-intolerant patients without severe adverse effects	NCCN, CSCO, ESMO	Targeted therapy, good tolerability
Cabozantinib	Second-line and third-line treatment, suitable for patients with disease progression after first-line therapy	NCCN, CSCO, ESMO	Multi-targeted kinase inhibitor
Ramucirumab	Second-line treatment, suitable for patients with AFP > 400 ng/mL and disease progression	NCCN, CSCO, ESMO	Specific inhibitor, high specificity
Camrelizumab	Second-line treatment, suitable for patients with advanced liver cancer and prior treatment failure	Chinese guidelines	Comparable efficacy to imported PD-1 inhibitors
Sorafenib	First/second-line treatment, suitable for patients unable to undergo surgery or local treatment	AASLD, EASL	Traditional targeted therapy, widely used
Lenvatinib	First/second-line treatment, suitable for patients with good liver function and without severe adverse effects	AASLD, EASL	Traditional targeted therapy, good tolerability
Durvalumab + Tremelimumab	First-line treatment, suitable for patients contraindicated for bevacizumab	AASLD	Dual immunotherapy, provides dual immune suppression
Surgical Resection	Suitable for BCLC 0-A stage patients, some BCLC B and C patients after multidisciplinary discussion	AASLD	Requires discussion in large liver centers
Liver Transplantation	Suitable for recurrent liver cancer patients meeting Milan criteria	AASLD, Chinese guidelines	Surgery preferred for recurrent liver cancer in China

Additionally, Chinese guidelines may emphasize the use of specific biomarkers and locally approved drugs, such as apatinib. Foreign guidelines typically recommend a broader array of diagnostic tools and systemic therapies, including advanced imaging techniques and a wider range of targeted and immunotherapy options. These differences underscore the importance of tailoring treatment strategies to regional practices and patient populations to optimize outcomes.

Recent advancements in liver cancer treatment have seen significant milestones, particularly in the first-line treatment. The National Comprehensive Cancer Network (NCCN) has made a groundbreaking inclusion of the atezolizumab and bevacizumab combination in its guidelines, marking the first approval of an immunotherapy combination for first-line treatment of liver cancer. Furthermore, the inclusion of Nivolumab as a monotherapy in the NCCN guidelines for first-line treatment underscores the expanding role of immunotherapy (173). Conversely, the European Society for Medical Oncology (ESMO) does not recommend chemotherapy as a first-line treatment, whereas Chinese guidelines still place high importance on it (174).

In the context of second-line treatment, the positions of the three major targeted therapies, such as regorafenib, cabozantinib, and ramucirumab, remain strong. However, significant controversy exists regarding the use of Nivolumab and pembrolizumab as second-line treatments. While NCCN and CSCO guidelines affirm the “Nivolumab and pembrolizumab combination” for second-line therapy, the Pan-Asian ESMO guidelines exclude K drug, and ESMO guidelines do not recommend either (173). The NCCN guidelines uniquely highlight the dual immunotherapy combination of nivolumab and ipilimumab as the first approved immunotherapy combination for second-line treatment. Additionally, the domestic drug camrelizumab has shown comparable efficacy to imported PD-1 inhibitors in second-line treatment of aHCC (174).

The AASLD recommends the use of serum AFP combined with ultrasound for liver cancer screening. Previously, the AASLD limited surgical resection indications to BCLC stage 0-A but now acknowledges that some BCLC stage B and C patients may be eligible for surgery following multidisciplinary discussion at large liver centers, particularly for patients with BCLC stage B and Vp1-Vp2 type portal vein tumor thrombus. For the first time, the AASLD also recommends adjuvant therapy post-surgery, currently advocating for the T+A regimen (175).

For recurrent liver cancer, liver transplantation is preferred if the patient meets the Milan criteria. However, in China, surgical resection remains the first choice for patients with recurrent HCC who are still eligible for surgery. For advanced liver cancer or intermediate liver cancer unsuitable for TACE, the AASLD recommends the T+A regimen as the first choice. For patients with contraindications to bevacizumab, the STRIDE regimen (durvalumab combined with tremelimumab) is recommended. For those contraindicated to immunotherapy, sorafenib or lenvatinib is recommended. In second-line treatment, the first choices are sorafenib or lenvatinib, previously used as first-line therapies (175).

In European guidelines, the T+A regimen is recommended as the first choice for systemic treatment-naive patients, with sorafenib or lenvatinib as alternative first-line options. Cabozantinib, regorafenib (for sorafenib-tolerant patients), and ramucirumab (for patients with AFP > 400 ng/mL) are recommended as second-line therapies following sorafenib treatment (176).

For tumor response evaluation, RECIST 1.1 is the preferred standard for assessing the response to systemic therapy. Other evaluative standards, such as immune-related RECIST and mRECIST, require further validation through prospective studies (176). Overall, the treatment guidelines for liver cancer in different regions reflect their respective clinical practices and cultural backgrounds, underscoring the importance of tailoring treatment strategies to regional circumstances and patient populations.

## Author contributions

LH: Data curation, Formal analysis, Investigation, Methodology, Writing – original draft. SL: Data curation, Formal analysis, Funding acquisition, Writing – original draft. FY: Writing – original draft. HW: Writing – original draft. YZ: Writing – original draft. XZ: Writing – original draft. XYH: Formal analysis, Funding acquisition, Writing – original draft. XPH: Formal analysis, Investigation, Validation, Writing – original draft.
